# The effect of music on comfort, pain, and anxiety in patients with bone marrow aspiration and biopsy in Turkey: a mixed-methods study

**DOI:** 10.1186/s12906-024-04531-0

**Published:** 2024-06-12

**Authors:** Seda Şahan, Emine Korkmaz, Serdal Korkmaz

**Affiliations:** 1 Department of Nursing Fundamentals, Faculty of Health Sciences, İzmir Bakırcay University, Menemen, İzmir 35100 Turkey; 2 Department of Nursing Fundamentals, Faculty of Health Science, Kırşehir Ahi Evran University, Kırşehir, Turkey; 3 Kayseri City Hospital, Hematology Clinic, Kayseri, Turkey

**Keywords:** Music, Bone marrow, Aspiration, Biopsy, Pain, Comfort, Anxiety

## Abstract

**Aim:**

This study was conducted to determine the effect of music on the pain, anxiety, and comfort levels of patients who underwent bone marrow aspiration and biopsy.

**Methods:**

This study was conducted on patients with hematological malignancies. Music was used with the intervention group. Patients’ pain, anxiety, and comfort levels were measured. In addition, qualitative data were obtained through in-depth interviews with patients.

**Results:**

A significant difference (*p* < 0.05) was found between the experimental and control groups regarding pain, comfort and anxiety levels following the application of music. It was found that there was a negative correlation between comfort and pain (*r*=-0.442 *p* < 0.001) and between comfort and anxiety (*r*=-0.544 *p* < 0.001). As a result of qualitative interviews, patients mentioned the relaxing effect of music and the reduction of anxiety and pain levels. They also stated that music can be utilized as an alternative method.

**Conclusion:**

According to the results of the present study, music reduced the pain and anxiety levels of the patients in bone marrow aspiration and biopsy and increased their comfort levels. We can say that music can be used in the clinic as a non-pharmacological method for pain, anxiety and comfort.

**Clinical Trials Number:**

NCT05895357 (Date:08/06/2023).

## Introduction

Bone marrow aspiration or bone marrow aspiration and biopsy (BMAB) are frequently performed in patients with hematological disorders to make an accurate diagnosis, determine the etiology of the disease, and ensure follow-up of the patients [1]. In adults, BMAB is a standardized procedure mostly performed under local anesthesia [2]. BMAB procedures are invasive procedures. Therefore, patients feel pain and anxiety during and after the procedure. It is one of the most painful procedures, especially for cancer patients [3]. Even if local anesthesia is applied before the procedure, patients feel pain during and after the procedure, and the duration and difficulty of the procedure are also associated with pain [4].

Yayla et al. (2019) reported that 55–70% of the patients undergoing these procedures described BMAB as a painful process [5]. The literature states that pain scores reported for BMAB range from 3.2 to 4 on a 10-point scale [2, 6]. At the same time, it was found that patients experience anxiety due to the pain experienced as a result of BMAB procedures [7]. It is stated that pain and anxiety have an effect that triggers each other during the BMAB procedure [3, 4]. A study reported that 60–80% of the patients experienced high levels of anxiety in BMAB procedures [8].

Pain and anxiety management is one of the fundamental aspects of nursing care. It is recommended to apply nursing interventions for pain and anxiety with non-pharmacological methods [7]. Music application, one of the most common and oldest non-pharmacological pain and anxiety management techniques is one of the non-pharmacological methods that can be used in BMAB [9]. Music has been used as an alternative method from past to present due to its physiological, psychological and social effects on people. At the same time, since it is non-invasive, accessible, inexpensive and easy to apply, it is frequently used in different patient groups [10, 11].

Discomfort is another predicament observed in BMAB. In a study, patients stated their discomfort level as 5.90 out of 10 after BMAB [12]. Comfort is one of the basic components of holistic nursing care. The studies on the comfort level of patients who underwent BMAB are rather limited. The number of studies examining the effect of music on pain and anxiety in BMAB is very few in the literature [13–15], while there is no study examining its effect on comfort level in BMAB.

Therefore, this study was carried out to determine the effect of music on the pain, anxiety, and comfort levels of patients who underwent BMAB. There is no previous study examining the effect of music on patients with hematological malignancies and its effect on comfort in bone marrow aspiration and biopsy. In addition, there is no qualitative study on the effects of music in bone marrow biopsy and aspiration procedures. For this reason, qualitative interviews were conducted to examine in depth the effects of music application on patients.

For this purpose, the following hypotheses were tested:

H1. Music reduces the anxiety level of patients undergoing BMAB.

H2. Music reduces the pain level of patients undergoing BMAB.

H3. Music increases the comfort level of patients undergoing BMAB.

## Methods

### Design

This research was conducted as a mixed methods study in the hematology polyclinic of a university hospital during the period of March to May 2023. This was a mixed-method study that used a segregated design with sequential synthesis using a two-phase approach [16]. Quantitative data was collected in the first phase of the research and qualitative data was collected in the second phase. In the first stage, quantitative data were found about the effect of music on patients. In this section, it was found that music was effective on pain, anxiety, and comfort. Thus, it allowed to obtain the qualitative data necessary to examine this effect in depth. Integration of the results occurred in the discussion in which we scrutinized quantitative and qualitative findings in relation to each other [16]. Qualitative interview results were analyzed through reflexive thematic analysis (RTA) as described by Braun and Clarke [17].This study was registered at the ClinicalTrials.org (register number: NCT05895357 Date:08/06/2023).

### Participants

The study was conducted with patients who were referred to the hematology clinic and who underwent BMAB for the first time. Inclusion criteria were as follows: to be 18 years of age or older, to undergo BMAB procedure, caring for an individual with hematological malignancy (defined as multiple myeloma, Chronic Lymphocytic Leukaemia, Myelodysplastic syndrome or indolent lymphoma)to have normal vital signs, to have adequate hearing, and to speak Turkish. Based on the exclusion criteria, those with hearing impairment and hormonal dysfunction (adrenal, pituitary, thyroid, etc.), those who were on anxiolytic and sedative drugs and who were diagnosed with severe anxiety disorder, Parkinson’s disease, Alzheimer’s disease, dementia, and major depression, were excluded from the study.

**Fig. 1:**
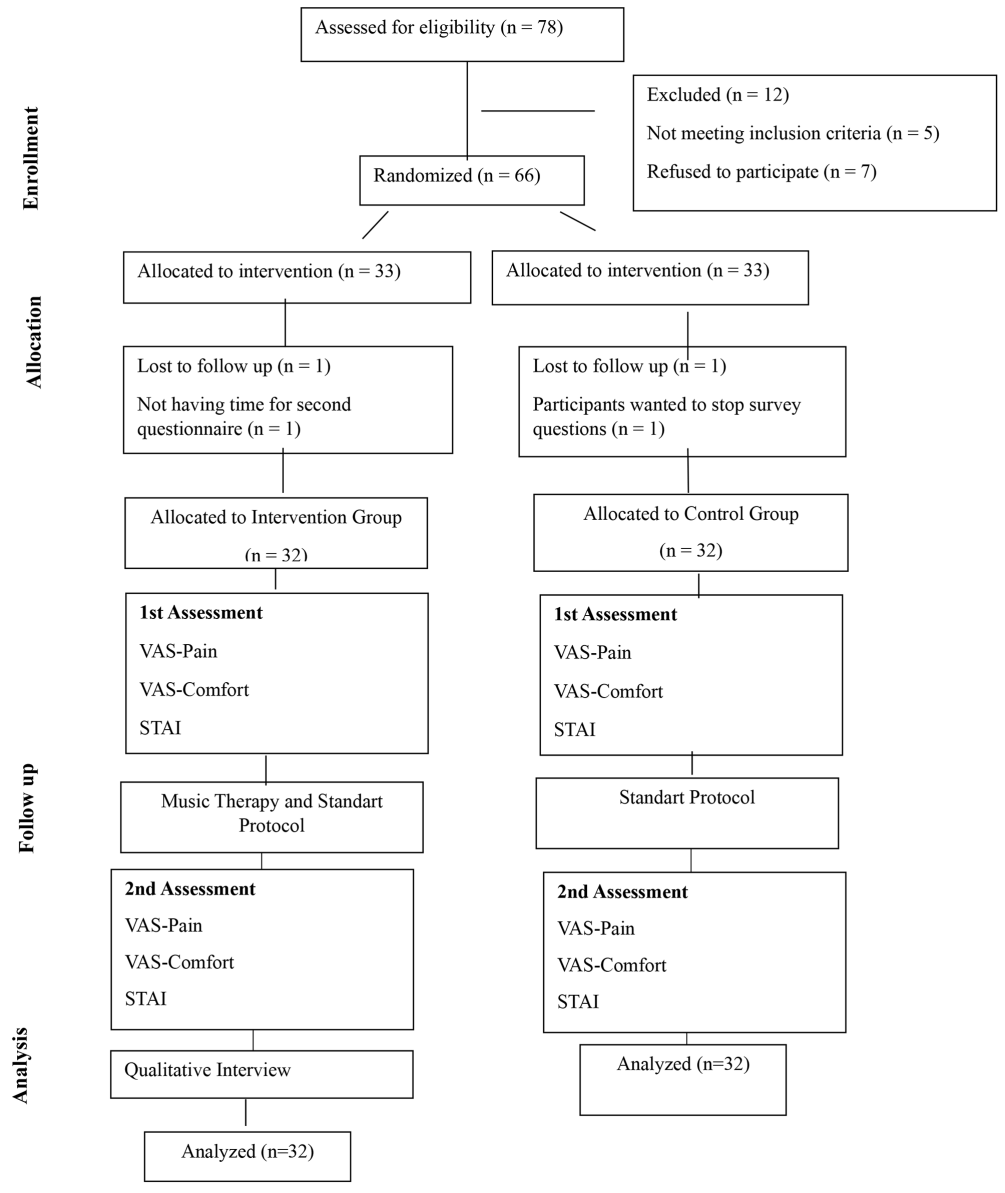
Consort Flow Chart

The sample group was calculated based on the effect size of other studies in the literature [11, 15]. According to the preliminary power analysis results using the G.Power 3.1.9 program, the sample size was calculated at 80% power, at medium effect size at 5% alpha value. According to the analysis results, the sample size per group was calculated as 30. However, since there was 10% probability that some patients may drop, the sample size for the study was recalculated as 66 (Experimental Group: 33, Control Group: 33). One patient in the experimental group did not fill in the post-intervention questionnaire saying that he did not have time, so the experimental group was completed with 32 patients. One patient in the control group did not want to answer the questions, the control group was completed with 32 patients as well (Fig. 1). Post hoc power analysis resulted in the following findings for the 32 control and 32 intervention groups: power (1-β) was 1.00 with α = 0.05 and effect size = 2.02 (according to t-test in independent groups for pain, anxiety and comfort scores).

### Randomization

The https://www.randomizer.org website was used to generate a random list of all patients who satisfied the inclusion criteria. On the website http://www.randomizer.org/, a statistician who was impartial and blind to the sample’s identity randomly assigned it to the intervention and control groups. Random selection was used to assign the participants; evens were assigned to the experimental group and odds to the control group. Due to the nature of the study, neither the researchers nor the patients could be blinded. However, this study was single-blind, as statistical analyses were performed by an independent researcher. After randomization, the patients in the intervention and control groups were homogeneous (Table 1).

### Data collection tools

#### Patient identification form

The form prepared by the researchers included patients’ age, gender and marital status [15, 18–20].

#### Visual analog scale (VAS)

Price et al. (1983) created the scale that is used to assess patients’ subjective levels of comfort and discomfort [21]. The pain and comfort thresholds on this scale are represented by the ends of a 10 cm horizontal or vertical line (0: no pain/no discomfort (extremely comfortable), 10: most severe pain/very uncomfortable (not at all). Patients described comfort as a pleasant emotion and the lack of physical pain. The patients were asked to rate their level of comfort and pain intensity on a scale. The individual’s pain score was determined by measuring the distance in millimeters between the stated location and the line’s lowest endpoint with a ruler.

#### State trait anxiety scale (STAI)

Spielberger et al. (1983) created the 40-item inventory [22]. The STAI consists of two separate scales to assess state (STAI-S) and trait (STAI-T) anxiety. Turkish research on the scale’s reliability and validity was carried out by Öner (1998) [23]. The STAI is made up of two distinct measures to measure both trait and state anxiety. This study made use of the State Anxiety Inventory, which contains 20 items (Items 1–20) that evaluate the respondent’s current emotions. The Likert scale ranges from 1 (Almost never) to 4 (Almost usually), with 4 being the highest rating. Scores on the subscale range from 20 to 80, with higher numbers indicating greater feelings of anxiety.

### Study protocol

One of the researchers contacted the patients who would undergo the BMAB procedure. Patients were informed about the purpose of the study and the procedure. Written and verbal consent was obtained from the patients who consented to participate in the study. Patients were then randomly assigned to the experimental and control groups. Patients in the experimental and control groups were instructed to fill out questionnaires about anxiety, comfort and pain before the BMAB procedure. There was no change in the standard treatment of the intervention and control groups during the study period. The data collection forms were filled before and after the procedure by the experimental and control group participants in a face-to-face meeting with the researchers.

### Intervention

Verbal and written consent forms were obtained from the patients who agreed to participate in the study. The patients were informed about the purpose of the study. Before the procedures, the patient’s pain, comfort, and anxiety levels were evaluated using VAS and STAI.

Because the response to music is generally individualized and dependent on the person’s prior experience and culture, allowing the participants to choose their favorite music is encouraged [24–26]. Kühlmann et al. (2018) and Chi and Young (2011) found that music chosen by the participants from a preselected list demonstrated positive outcomes [24, 25]. As a result, listening to patient-preferred music has been shown to be beneficial for reducing anxiety and pain [27, 28]. For this reason, in consultation with the music therapist, it was preferred that the patients listen to music of their own choice in this study. Before starting the procedure, the patients were asked to choose the non-verbal (without lyrics) music they wanted to listen to. The music selected by the patients was evaluated in terms of rhythm. A systematic review on music therapy revealed that music with a tempo between 60 and 90 bpm and non-verbal music was more effective in relaxation and stress reduction [27].

After appropriate music was selected, the patients were admitted to the procedure room. The music was played through a speaker (JBL Flip). Patients were informed about the buttons to turn the music on-off and adjust the volume. Patients were informed that they could turn off the music if they felt uncomfortable during the procedure or if they wanted to stop. At the same time, they were also informed that they could lower or raise the volume of the music, as the volume of the music could vary according to their hearing. Patients were asked to report any discomfort they felt during the music recital. However, there was no patient in the intervention group who did not want to listen to the music or stopped listening to the music. Patients kept listening to the same music throughout the BMAB procedure.

After the procedure was finished, the music player was turned off. The patients were asked if they felt any discomfort while the music played. The patients reported no discomfort or concerns concerning the use of music. Music application lasted an average of 30 min. After completing the procedures, the patient’s pain, comfort, and anxiety levels were re-evaluated using VAS and STAI. Data were collected simultaneously.

The patients in the control group did not listen to music, and standard clinic procedures were completed without any further involvement. Before and after the procedure, participants in the control group were assessed for anxiety, pain, and comfort.

### Qualitative interview

Individual interviews were conducted one-on-one with the patients after the music application was finalized. Qualitative interviews were conducted by another researcher who did not perform the music to avoid bias. Qualitative interviews were conducted with 18 patients who agreed to participate in the interview. The interviews were conducted in an empty, quiet room in the clinic after the music intervention.

In the interview form utilized for the qualitative interview, open-ended questions were addressed to determine the patients’ attitudes toward music and the effect of music on the patients. In this way, questions that could not be answered or clarified as a result of the primary data (quantitative part) are provided with secondary data (qualitative interview) [29]. In this study, both the primary and secondary data processes fundamentally have the same structure and are based on the effect of music. At the same time, with qualitative interviews, it is aimed to find answers to questions that cannot be reached in quantitative interviews. With qualitative interviews, participants express their feelings and thoughts freely [30].

In the qualitative interview, patients were asked, ‘’What were the positive and negative effects of music for you?‘’ ‘’What emotions did you experience during music ?‘’, ‘’What did music mean to you?‘’ ‘’Would you like to use music in other procedures?‘’, ‘’What were your opinions about music application before the intervention and how/were your opinions changed currently?‘’. During the questions, guidance such as “What did you mean?” and “Can you explain a little more?” was provided so that the patients could elaborate more. The qualitative interview with the patients lasted an average of 60 min.

### Data analysis

#### Quantitative data analysis

The SPSS (Statistical Package for Social Science) 21.0 package program was used to analyze the research data. The Shapiro-Wilk normality test was used to determine the normality distribution of the data. Descriptive statistics were given as the mean ± standard deviation and the median minimum value and maximum value. In the categorical variable comparisons, the chi-squared exact test was used, whereas the Mann-Whitney U test was used for the independent two-group comparisons and the Wilcoxon test for the dependent within-group comparisons. Furthermore, the effect size was employed to detail the comparative results. Cohen d was used to compute effect sizes. The effect size was classified as small (d = 0.20), medium (d = 0.50), and large (d = 0.80)(Cohen, 2013). Correlation analysis was employed for post-test comparisons of patients’ pain, comfort and anxiety. Consultancy was received from a biostatistician for the quantitative data of the study.

#### Qualitative data analysis

Audio recordings were recorded during the qualitative interviews with the patients. At the same time, the researcher took notes on a piece of paper for any questions that needed attention or guidance. The audio recordings of the interviews were transcribed by a researcher. The qualitative data of the study were analyzed using the 6 steps established by Braun and Clark [17].

1. Step: Two researchers repeatedly read the transcribed audio recordings. The written text was transferred to MAXQDA 20.0.
2. Step: At this stage, similarities and differences in the statements of the patients were recorded according to the first impression. Then, coding was carried out for each relevant text Sect. 
3. Step: The codes created in the 2nd stage were re-evaluated by the researcher and divided into more precise and descriptive codes. At this stage, the codes were inputted into the MAXQDA system separately.
4. Step: Potentially interesting text passages coded in the previous stage were re-read by the authors. The text was discussed until the authors agreed on a common decision. The authors then developed preliminary themes. The codes were compared with other codes within the same cluster to determine whether the ongoing ranking was appropriate considering the purpose and data of the study. In this way, it was evaluated for which preliminary themes the codes were more appropriate.
5. Step: Small changes were implemented in the ordering of the coded data among the themes. At this stage, the researchers prepared small summary texts about the content of these themes. In this way, it was revealed which codes did not belong to that theme, which ones yielded better results in another theme, and reordering was performed.
6. Step: for each theme and sub-theme, clear definitions were provided in relation to the literature.

## Results

### Quantitative results

Table 1 presents participants’ descriptive characteristics. When the descriptive characteristics of participants in the experimental and control groups were compared, no statistically significant difference was found between the groups (*p* > 0.05, Table 1).

**Table 1:** Distribution of the introductory characteristics of subjects in the intervention and control groups

	Experimental Group (*n* = 32)		Control Group (*n* = 32)		Test
Age	50.70 ± 13.89		52.34 ± 14.67		t = 0.003 *p* = 0.567
Gender	n	%	n	%	
Female	17	53.2	19	59.3	x^2^ = 0.513 *p* = 0.358
Male	15	46.8	13	40.7	
Marital status					
Married	24	75.0	27	84.4	x^2^ = 0.285 *p* = 0.427
Single	8	25.0	5	15.6	
Hematological disease					
ALL	7	21.8	8	25.0	x^2^ = 0.427 *p* = 0.628
AML	20	62.4	19	59.3	
CML	1	3.2	1	3.2	
MS	2	6.3	3	9.3	
MM	2	6.3	1	3.2	

ALL: Acute Lymphoblastic Leukemia, AML: Acute Myeloid Leukemia, CML: Chronic Myeloid Leukemia, MS: Myelodysplastic Syndrome, MM: Multiple Myeloma

A statistically significant difference was found between the experimental and control groups in the STAI level after the application of music (*p* = 0.001). The intergroup effect size was found to be above the mean after the intervention (d = 0.63 CI 2.966–9.316) (Table 2).

**Table 2:** Comparing the mean scores for anxiety between the study groups (*n* = 32)

Group	T0		T1		Test Statistic^b^	d	95% CI
	X̄±SD	X̃[IQR]	X̄±SD	X̃ [IQR]			
Experimental Group	44.06 ± 7.65	38 [8]	30.13 ± 9.70	15.5 [6]	*p* = 0.001**	0.63	2.966–9.316
	*p* = 0.008^a**^						
Control Group	43.86 ± 6.29	38 [13]	38.46 ± 15.83	48 [8]			
	*p* = 0.074^a^						

a = Wilcoxon test b = Mann–Whitney U, p**: *p* < 0.05 Significance level, d: Effect size for Cohen d, X : Average, SD : Standard Deviation, X̃ : Median, IQR: Interquertile Range, T0 : First Measurement, T1: Final Measurement.

There was a significant difference in pain levels between the experimental and control groups after the application of music (*p* = 0.021). After the intervention, the intergroup effect size was found to be above the mean (d = 0.53 CI 0.422–1.910) (Table 3).

**Table 3:** Comparing the pain mean scores between the study groups (*n* = 32)

Group	T0		T1		Test Statistic^b^	d	95% CI
	X̄±SD	X̃[IQR]	X̄±SD	X̃ [IQR]			
Experimental Group	3.53 ± 1.29	3 [1]	2.61 ± 1.54	2[1.5]	*p* = 0.021**	0.53	0.422-1.910
	*p* = 0.021^a **^						
Control Group	3.72 ± 1.34	3 [2]	3.50 ± 1.79	3 [3]			
	*p* = 0.266^a^						

a = Wilcoxon test b = Mann–Whitney U, p**: *p* < 0.05 Significance level, d: Effect size for Cohen d, X : Average, SD : Standard Deviation, X̃ : Median, IQR: Interquertile Range, T0 : First Measurement, T1 : Final Measurement.

There was a significant difference in comfort levels between the experimental and control groups after the application of music (*p* = 0.001). After the intervention, the intergroup effect size was found to be above the mean (d = 0.53 CI 0.422–1.910) (Table 3). The effect size between groups after the intervention was very high (d = 1.27 CI 3.125–4.984) (Table 4).

**Table 4:** Comparing the mean scores for comfort between the study groups (*n* = 32)

Group	T0		T1		Test Statistic^b^	d	95% CI
	X̄±SD	X̃[IQR]	X̄±SD	X̃ [IQR]			
Experimental Group	4.11 ± 1.75	3 [2]	7.83 ± 1.86	7[2.5]	*p* = 0.001**	1.27	2.215-5.984
	*p* = 0.001^a **^						
Control Group	4.56 ± 2.19	4 [2]	5.73 ± 1.39	4 [1]			
	*p* = 0.196^a^						

a = Wilcoxon test b = Mann–Whitney U, p**: *p* < 0.05 Significance level, d: Effect size for Cohen d, X : Average, SD : Standard Deviation, X̃ : Median, IQR: Interquertile Range, T0 : First Measurement, T1 : Final Measurement.

According to the post-test results of the experimental group, it was found that there was a negative correlation between comfort and pain (*r*=-0.442 *p* < 0.001) and between comfort and anxiety (*r*=-0.544 *p* < 0.001). It was found that a positive and significant correlation between pain and anxiety (*r* = 0.441 *p* < 0.001) (Table 5).

**Table 5:** The relationship between pain, anxiety and comfort scores in the intervention group

	Comfort	Anxiety	Pain
Comfort	-	*r*=-0.544 *p* < 0.001	*r*=-0.442 *p* < 0.001
Anxiety			*r* = 0.441 *p* < 0.001

### Qualitative interview results

#### Theme 1. the effects of music

Patients were interviewed about the effects of music. All of the interviewed patients mentioned that music is positive, relaxing and reduces pain. In addition, the patients did not mention any negative effects of music. When talking about the effects of music, especially patients frequently mentioned the expressions “anxiety relieving”, “pain relieving”, “relaxing”.

#### Sub-Theme 1. relaxing effect of music

Many of the patients who received music mentioned that music relaxed their anxiety during the procedure.

‘’I had this procedure for the first time and I was really scared before the procedure. This situation scared me a lot. But as soon as the music started to play, I just closed my eyes and focused on the music. The music reduced my anxiety and I felt very comfortable during the procedure. Both my worries decreased and I really think that for the first time, the procedure was performed comfortably in the hospital.’’(Patient 4).

‘’It was really exhausting and upsetting to think not only about the process but also about how my results would turn out. These were the only things on my mind. But then I started listening to the music and I remember my hands stopped shaking and sweating. Listening to music was very helpful. ‘’ (Patient 7,11).

#### Sub-Theme 2. the positive distraction effect of music

Patients reported that music allowed them to focus for a moment on life and emotions outside the hospital. Patients have reported that music alleviated the ‘anxiety-inducing’ effect of BMAB procedure and the feeling of being in the hospital. Some patients also stated that they felt that the music was playing in their ears all day. They mentioned that they would like to use music regularly as it makes hospital procedures more “bearable”.

‘’It’s an incredible feeling to get caught up in the rhythm of the music and relax. For a moment I forgot I was in the hospital, I was thinking about completely different things. Sometimes I imagined myself in front of a panorama…’’ (Patient 12).

‘’Towards the middle of the music, I couldn’t recognize the smell of the hospital, I took a deep breath.’’ (Patient 9).

#### Sub-Theme 3. the ‘’pain relieving’’ Effect of Music

Patients mentioned that the music prevented them from focusing on the procedure and shifted them in a different direction. They mentioned that they did not focus on the feeling of pain, especially with music, and that their pain levels decreased by focusing on other emotions and situations in their minds.

“I was most worried about how much pain I would feel. But the music really helped me a lot. As I listened to the music, I remembered memories of being happy before. After a while, I started humming the music and felt little pain.‘’ (Patient 14).

“After listening to music, I wished I had done this before for all the procedures I was in pain… I felt comfortable, like I was not in the hospital… I would prefer to listen to music when I come to the hospital from now on.’’ (Patient 1).

‘’ During the procedure I just listened to the music and felt it, so I felt less pain during the procedure, which was a very big thing for me.’’ (Patient 2).

‘’Listening to music reduced my focus on my pain. That’s why I didn’t feel pain, I just listened to music and relaxed.’’ (Patient 16).

#### Theme 2. attitude towards music

It was observed that there were differences in the attitudes of patients towards music. Some patients expressed their willingness for music by stating that they listened to music to get away from the stress of daily life. However, some patients mentioned that it was “confusing” to listen to music when they were “anxious” enough to worry about the procedure.

#### Sub Theme 1. music in everyday life

Patients mentioned that they often listen to music in daily life. However, they mentioned that the hospital environment is now a “part of daily life” for them, and they did not experience this in the hospital.

‘‘I usually listen to music every day, when I go to work, when I work at home, when I drive. Music relaxes me and makes me feel good, but I had never listened to music before when I was in the hospital. I was skeptical at first, but after observing the effect of music when I was in the hospital, I’m glad I did.‘’ (Patient 15).

‘’Unfortunately, being in hospital is part of my daily life for a long time and this process also continues at home. It was the first time I listened to music during a procedure, and I enjoyed it. I didn’t even realize how quickly the procedure was over.’’. (Patient 6).

‘’ I definitely want to listen to music in my future hospital experiences. I felt really good. Just focusing on the music relaxed me.’’ (Patient 3–8).

#### Sub-Theme 2. proposing music as an alternative method

All patients reported that this was the first time they had listened to music in hospital or during a procedure. Patients described music as a positive experience. And they mentioned that they wish they could experience it all the time they are in the hospital.

‘‘I have to come to the hospital for many procedures, diagnoses and treatments. Each visit triggers a different anxiety for me. After listening to the music today, I simply thought, ‘I should do this all the time.’’ (Patient 10,18).

“Every time I come to the hospital I want to listen to this music and get away from my pain. I think it really helps. I believe all patients should be provided with this opportunity.’’ (Patient 5).

‘’ In hospitals, especially during such painful procedures, patients should be advised to listen to music. I think everyone will relax when they listen to the music they want. I think it is a completely different dimension to be able to help the patient in this way.’’ (Patient 17).

## Discussion

This study investigated the effect of music on pain, anxiety and comfort level during BMAB. According to study results, music reduced patients’ pain and anxiety levels and increased their comfort.

Music has a distracting and supportive effect. Especially in short-term procedures, music has a significant distraction effect [25]. Ergin et al. (2022) found that music significantly reduced anxiety in patients in the intervention group who underwent BMAB [14]. In the pilot study, Vestegern et al. (2018) stated that music significantly reduced anxiety in patients with bone marrow aspiration [31]. In their study with cancer patients, Geyik et al. (2021) reported that music reduced anxiety [32]. Contrary to these studies, in their study Özdemir et al. (2019) stated that music applied to patients during bone marrow aspiration did not have a positive effect on patients but increased their anxiety levels [15]. The present study concluded that music applied to patients during BMAB procedure reduced anxiety. Effect size analysis was also performed in the present study. According to the results of this analysis, it was found that music had an above-average effect on anxiety. Conducting the effect size analysis presented the strong side of the study results in the present study. It can be argued that the post-procedure anxiety of the patients was further reduced thanks to the distraction feature of the music. It is possible to say that the patients’ anxiety decreased in the present study, especially since music increased the release of endorphins, ensured the emergence of positive emotions, and reduced negative emotions such as fear and anxiety.

According to the qualitative interview results, patients described music as “relaxing” and “distracting”. They mentioned that listening to music, especially during the procedure, reduced their anxiety about the procedure. They also stated that music reduced trembling and sweating, which are symptoms of anxiety. In this case, all of the patients reported that music had positive effects on anxiety. The qualitative findings of the study also indicate similar results with the quantitative findings of the study.

Music works through distraction by competing for attention from a negative stimulus and ultimately producing pleasure [33]. By reducing the serum cortisol levels by acting on the frontal cortex, it alleviates perceived pain and causes mental relaxation, ultimately increasing patient comfort, coping ability, and sense of well-being [15, 33]. In addition, music increases patient satisfaction by helping time pass faster via distraction [7]. Although complication rates are low in bone biopsy/aspiration procedures, patients experience pain [34]. The literature shows that reported pain scores for BMAB range from 3.2 to 4 on a 10-point scale [2, 6]. In their study, Jaddini et al.(2016) reported that patients’ pain levels were high during the BMAB procedure [4]. Ergin et al. (2022) found that classical Turkish music played for patients who underwent BMAB significantly reduced pain compared to the control group [14]. Similarly, the present study determined that music decreased pain in BMAB with a statistically significant difference in the experimental group. In addition, a significant difference was found between the experimental and control groups. It was concluded that music reduced pain with an effect size above the average. According to the study results obtained in the present study, music reduced pain in BMAB. Especially since the effect size was found to be above average, it can be suggested to use music non-pharmacologically in BMAB procedures. After the qualitative interview with the patients, they stated that music reduced their anxiety and at the same time reduced their pain levels. One patient interviewed even described music as a “painkiller”. Patients also mentioned in their statements that they had anxiety about feeling pain. For this reason, some patients stated that there was a decrease in the level of pain with music and accordingly a decrease in their anxiety. Especially during music listening, we can state that the patient does not focus on their pain as they tend to think about a different emotion or situation other than the procedure and thus they experience less pain.

Nursing care is based on approaching patients holistically. Comfort, a basic need, is one of the basic elements of holistic nursing care [18]. Comforting the patients about the procedures to be performed is one of the responsibilities of the nurses. Music is one of the methods that are used to increase patients’ comfort [20]. Bone marrow biopsy/aspiration procedures are invasive; bone marrow samples are obtained from the iliac crest, anterior iliac crest, or sternum, typically using a needle. Therefore, bleeding, pain, and discomfort may be seen in patients after the invasive intervention [1]. In one study, 59% of patients reported moderate discomfort during bone marrow aspiration [35]. A study conducted by Karadağ et al. (2019) found that the intervention by using listening to music for women with breast cancer during radiation therapy significantly increased their comfort levels [20]. In the present study, it was found that the comfort level of the experimental group that received music increased significantly after the intervention while there was no significant increase in the control group. In addition, a statistically significant difference was found between the experimental and control groups after the intervention. According to the effect size analysis, music increased comfort with a large effect during BMAB. Considering the qualitative interviews, some patients mentioned that they had a more “comfortable” procedure process due to the relaxing effect of music. In particular, we can state that the decrease in the pain levels of the patients due to music and the decrease in their anxiety increased their comfort levels.

According to the results of the qualitative interviews with the patients, the patients suggested that music should be practiced not only for the BMAB procedure but also for all diagnosis, examination and treatment procedures conducted in the hospital. Especially some of the patients visit the hospital constantly due to their medical condition. For this reason, some of the patients stated that they would like to receive music every time they come to the hospital. At the same time, the patients stated that music should be implemented on different patients, so that each patient can experience music. They stated that music is an effective “alternative” therapy method for patients.

Likewise, according to the qualitative interview results, some patients stated that they felt the effect of music in their daily lives. At the same time, some patients stated that hospital visits were part of daily life for them, so they were glad to experience music. While some patients had prejudiced/negative attitudes towards music therapy, they stated that they realized the effects of music after the intervention and that they were very pleased. Accordingly, we can claim that music has positive effects on patients and can be recommended as an alternative method for symptoms in patients.

Correlation analysis was performed for the comfort, pain and anxiety levels of the experimental group. According to the results of the analysis, a negative relationship was found between comfort and pain, and between comfort and anxiety. According to this result, the comfort of the patient increases as the pain and anxiety decrease. With the application of music, the pain and anxiety levels of the patients are reduced in BMAB application, and accordingly, there is an increase in their comfort level. No study was found in the literature on the effect of music on comfort during BMAB procedures. It is believed that the lack of research in this issue constitutes a gap in the literature. BMAB procedure takes an average of 15–20 min. The results of this study and the findings presented in the literature show that patients’ anxiety levels before the procedure were very high while their comfort levels were low. Therefore, it can be argued that music is an effective method to increase patients’ comfort levels during the procedure.

## Conclusion

According to the results of this study, music decreased pain and anxiety in BMAB procedures while it increased the comfort level. In the post-test results of the experimental and control groups, it was observed that there was a significant difference in pain, anxiety and comfort levels. It was determined that music reduced anxiety and pain and increased comfort level in patients who underwent BMAB procedure. At the same time, with qualitative interviews, the perceptions of the patients towards music were examined precisely. According to the qualitative interviews, the patients mentioned only positive effects of music. Patients stated that music should be practiced in every patient group and they recommended it to be utilized as an alternative method.

## Limitations

This study was performed in a single center, in a single hematology outpatient clinic. For this reason, it is suggested to carry out further research in different centers.

## Data Availability

The datasets used and/or analysed during the current study are available from the corresponding author on reasonable request.
